# A Time Delay Neural Network Based Technique for Nonlinear Microwave Device Modeling

**DOI:** 10.3390/mi11090831

**Published:** 2020-08-31

**Authors:** Wenyuan Liu, Lin Zhu, Feng Feng, Wei Zhang, Qi-Jun Zhang, Qian Lin, Gaohua Liu

**Affiliations:** 1 School of Electronic Information and Artificial Intelligence, Shaanxi University of Science and Technology, Xi’an 710021, China; liuwenyuan@sust.edu.cn; 2 School of Control and Mechanical Engineering, Tianjin Chengjian University, Tianjin 300384, China; zeal286@163.com; 3 Department of Electronics, Carleton University, Ottawa, ON K1S 5B6, Canada; fengfeng@doe.carleton.ca (F.F.); qjz@doe.carleton.ca (Q.-J.Z.); 4 College of Physics and Electronic Information Engineer, Qinghai University for Nationalities, Xining 810007, China; linqian@tju.edu.cn; 5 School of Electronics and Information Engineering, Tianjin University, Tianjin 300072, China; suppig@126.com

**Keywords:** nonlinear device modeling, neural networks, optimization methods

## Abstract

This paper presents a nonlinear microwave device modeling technique that is based on time delay neural network (TDNN). The proposed technique can accurately model the nonlinear microwave devices when compared to static neural network modeling method. A new formulation is developed to allow for the proposed TDNN model to be trained with DC, small-signal, and large signal data, which can enhance the generalization of the device model. An algorithm is formulated to train the proposed TDNN model efficiently. This proposed technique is verified by GaAs metal-semiconductor-field-effect transistor (MESFET), and GaAs high-electron mobility transistor (HEMT) examples. These two examples demonstrate that the proposed TDNN is an efficient and valid approach for modeling various types of nonlinear microwave devices.

## 1. Introduction

Artificial neural network (ANN) is a recognized tool for modeling and design optimization in RF and microwave computer-aided design (CAD) [1,2,3,4,5,6,7,8,9]. This technique has been successfully used in parametric modeling of microwave components [10,11,12], electromagnetic (EM) optimization [13,14], parasitic modeling [15], nonlinear device modeling [16,17,18], nonlinear microwave circuit optimization [19,20,21,22], power amplifier modeling [23,24,25], and more.

This paper addresses the nonlinear device modeling area. Nonlinear device modeling is an important area of CAD and a variety of device models have been built. With the rapid development of semiconductor industry, new devices constantly evolve. The existing models may not be accurate for the new devices. Therefore, there is an ongoing need for new models. The challenge for CAD researchers is not only to develop new models, but also to introduce new CAD methods.

Traditionally, the equivalent circuit modeling approach is a vital modeling technique for nonlinear device modeling. The existing equivalent circuit models need to be modified in order to fit for different devices. The parameters in the equivalent circuit need repetitively changes and sometimes the parameters are mutually contradictory. Especially, when it comes to a new device, it is time consuming to build a nonlinear model that is based on equivalent modeling technique. For an alternative approach, the ANN model can be an efficient trained and implemented model [1], which can be systematically developed by neural network training process. ANN technique recently can be used to approach device modeling problem with good accuracy. When the nonlinear and dynamic effects in the device become significant, we need more advanced neural network-based techniques to approach device modeling problem. Several different types of neural networks, such as dynamic neural networks (DNNs), real-valued time-delay neural networks (RVTDNNs), and recurrent neural networks (RNNs), have been used for nonlinear circuit modeling [20,21,22]. These neural network-based techniques are more flexible to build more general models. Recently, the dynamic neuro-space mapping technique [26] can also deal with the nonlinear device modeling problem well, which contains coarse model and neural network. However, building a proper coarse model needs repetitive changes of the parameters in equivalent circuits.

In this paper, we focus on directly modeling methods that can systematically establish models without building proper equivalent circuit models. In this paper, we propose a time delay neural network (TDNN) technique for nonlinear microwave device modeling using DC, small-signal, and large-signal information for the first time. A new formulation to train the proposed TDNN with DC, small-signal, and large signal data is proposed. An algorithm to train the proposed TDNN model is formulated. Examples of GaAs metal-semiconductor-field-effect transistor (MESFET) and GaAs high-electron mobility transistor (HEMT) modeling is used to demonstrate the validity of the proposed TDNN method.

## 2. Formulations of the Proposed Time Delay Neural Network (TDNN) Model

According to a nonlinear device, $u={\left[\begin{array}{cccc} u_{1} & u_{2} & ... & u_{m} \end{array}\right]}^{T}$ represents the vector of the input signals, while $o={\left[\begin{array}{cccc} o_{1} & o_{2} & ... & o_{N_{o}} \end{array}\right]}^{T}$ are the output signals, where m is the number of the inputs and *N_o_* is the number of outputs. For example, $u={\left[\begin{array}{cc} v_{g} & v_{d} \end{array}\right]}^{T}$ and $o={\left[\begin{array}{cc} i_{g} & i_{d} \end{array}\right]}^{T}$ in the transistor example, where m=2 and $N_{o}=2$. In this example, *v_g_* and *v_d_* represent the gate voltage and the drain voltage, respectively. *i_g_* and *i_d_* are the gate current and the drain current, respectively. Let $f_{ANN}$ represent the multilayer neural network. ***w*** represents the internal weight of the neural network. The general TDNN equation in time domain can be used in order to describe the original nonlinear device as

(1) $$o\left(t\right)=f_{ANN}\left(u\left(t\right),\;u\left(t-\tau \right),\;\;\ldots ,\;u\left(t-N_{d}\tau \right),\;w\right)$$

where τ is a time delay parameter and $N_{d}$ represents the total number of delay steps.

**Figure 1:**
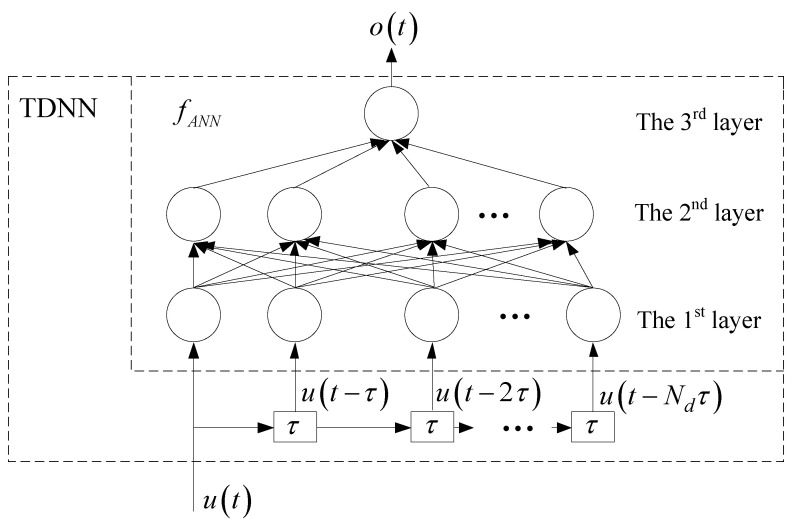
The structure for time delay neural network (TDNN).

Suppose that the TDNN model contains one input and one output and the $f_{ANN}$ is a three-layer multilayer perceptron (MLP) model. Therefore, Figure 1 shows the TDNN structure. In this figure, the TDNN structure contains external delay information compared with MLP model.

In this paper, the $f_{ANN}$ of the TDNN is a three-layer MLP. The first layer of the MLP is the input relay layer, the second layer is the hidden layer, and the third layer is the output layer. The sigmoid function is used as the activation function in the internal hidden layer.

After the neural network well trained by the device data, the TDNN model can be a good model. We can usually get DC, S-parameters, and harmonic data for nonlinear device modeling from measurement or simulation. Therefore, we propose an analytical formulation of TDNN for nonlinear device modeling using DC, bias-dependent S-parameter data, and large-signal harmonic balance (HB) data.

Let U represent the DC input signals and O be DC output signals. Therefore, the delayed signals of inputs in DC condition are all equal to U. The output of TDNN in DC case is derived as

(2) $$O=f_{ANN}\left(\overset{N_{d}+1}{\overset{︷}{U,\;\;U,\;\;...,\;\;U}},\;\;w\right)$$

Let Y represent the small signal transfer function of the system. In transistor example, matrix Y represents Y-parameters. Let $U_{bias}$ denote the DC bias of u. The small-signal S-parameters are derived through the Y-parameters of the TDNN model that are shown in Equation (3). In Equation (3), the derivative of $f_{ANN}$ can be obtained using the adjoint neural network method [27], and *k* represents the index of delay buffers. The Y matrix, defined as the sum of products of $e^{-j\omega k\tau }$ and $\partial f_{ANN}/\partial u$ in (3), is frequency dependent due to the use of delayed signal in output function $f_{ANN}$. Hence, the proposed TDNN model is a non-quasi static (NQS) model [28,29,30,31], when $N_{d}>0$. In Equation (3), jω=j2πf, where f represents frequency.

(3) $$Y={\left(\sum_{k=0}^{N_{d}}e^{-j\omega k\tau }\cdot {\frac{\partial f_{ANN}^{T}\left(u\left(t\right),\;u\left(t-\tau \right),\;\;\ldots ,\;u\left(t-N_{d}\tau \right),\;w\right)}{\partial u\left(t-k\tau \right)}|}_{u\left(t\right)=\;u\left(t-\tau \right)=\;\ldots =u\left(t-N_{d}\tau \right)=U_{bias}}\right)}^{T}$$

In the large-signal case, suppose the generic harmonic frequency be $\omega_{k}$, where the subscript k represents the index of harmonic frequency $k=0,1,2,...,N_{H}$. $N_{H}$ is the number of harmonics that are considered in HB simulation. $N_{T}$ represents the number of time points. Let $W_{N}\left(n,k\right)$ denote the Fourier coefficient for nth time sample and the kth harmonic frequency, where *n* = 1,2,…,*N_T_* and *k* = 1,2,…,*N_H_*. Let superscript* represent complex conjugate. Let $U\left(\omega_{k}\right)$ and $O\left(\omega_{k}\right)$ be the input and output signals in the frequency domain, respectively. Given input $U\left(\omega_{k}\right)$ for all k, $u(t_{n}-K_{\tau })$ can be computed from Equation (4), where *K =* 0, 1, 2, …, *N_d_*. The outputs $O\left(\omega_{k}\right)$ are computed as in Equation (5). The frequency domain delay functions $e^{-j\omega_{k}\tau }$, $e^{-j\omega_{k}2\tau }$,…,$e^{-j\omega_{k}N_{d}\tau }$ are introduced into the training equation. The proposed technique can accurately model the nonlinear behavior of the device by training the TDNN model with DC, S-parameter, and HB data.

(4) $$u\left(t_{n}-K\tau \right)=\sum_{k=0}^{N_{H}}U\left(\omega_{k}\right)W_{N}^{*}\left(n,k\right)e^{-j\omega_{k}K\tau }$$

(5) $$O\left(\omega_{k}\right)\;=\frac{1}{N_{T}}\sum_{n=0}^{N_{T}-1}f_{ANN}\left(u\left(t_{n}\right),\;\;u\left(t_{n}-\tau \right)\;,...,u\left(t-N_{d}\tau \right)\right)\;\cdot W_{N}\left(n,k\right)\;\;$$

We systematically described above the TDNN model equation used in DC, small-signal, and large-signal simulation. Because of the neural network universal approximation capability [1], such TDNN model can achieve satisfied accuracy.

## 3. An Algorithm for Training the Proposed TDNN Model

Our proposed TDNN model will be good after the neural networks being well trained by DC, S-parameters, and HB data of the nonlinear device. The training error is formulated as

(6) $$\begin{array}{ll} E_{Tr}\left(w\right) & =\alpha E_{DC}\left(w\right)+\beta E_{S}\left(w\right)+\gamma E_{HB}\left(w\right)\\ & =\alpha \left(\frac{1}{2}\sum_{k\in T}{\left\|O\left(x_{k},w\right)-O_{k}^{\left(d\right)}\right\|}^{2}\right)\;+\beta \left(\frac{1}{2}\sum_{k\in T}{\left\|S\left(x_{k},w\right)-S_{k}^{\left(d\right)}\right\|}^{2}\right)\;\;+\gamma \left(\frac{1}{2}\sum_{k\in T}{\left\|HB\left(x_{k},w\right)-HB_{k}^{\left(d\right)}\right\|}^{2}\right) \end{array}$$

where *E_Tr_* represents the total training error, *E_DC_* represents the error between DC responses of the proposed TDNN model and the DC device data, *E_S_* represents the error between small-signal responses of the proposed TDNN model and the small-signal device data, and *E_HB_* represents the error between large-signal responses of the proposed TDNN model and the large-signal device data. α, β  and γ  represent the weighting factors for DC error *E_DC_*, small-signal error *E_S_*, and large-signal error *E_HB_*, respectively. The weighting factors α, β  and γ  can be roughly determined by the value range of the training data and the number of DC data, small-signal data, and large-signal harmonic data. ***O***(.), ***S***(.) and ***HB***(.) represent the DC, bias-dependent S-parameters and HB response of the proposed TDNN model, respectively. $O_{k}^{\left(d\right)}$, $S_{k}^{\left(d\right)}$ and $HB_{k}^{\left(d\right)}$ represent the *k*th training data of DC, bias-dependent S-parameters and HB, respectively. *T* represents of training sets. We use real and imaginary types of the HB data for training in the proposed TDNN technique.

**Figure 2:**
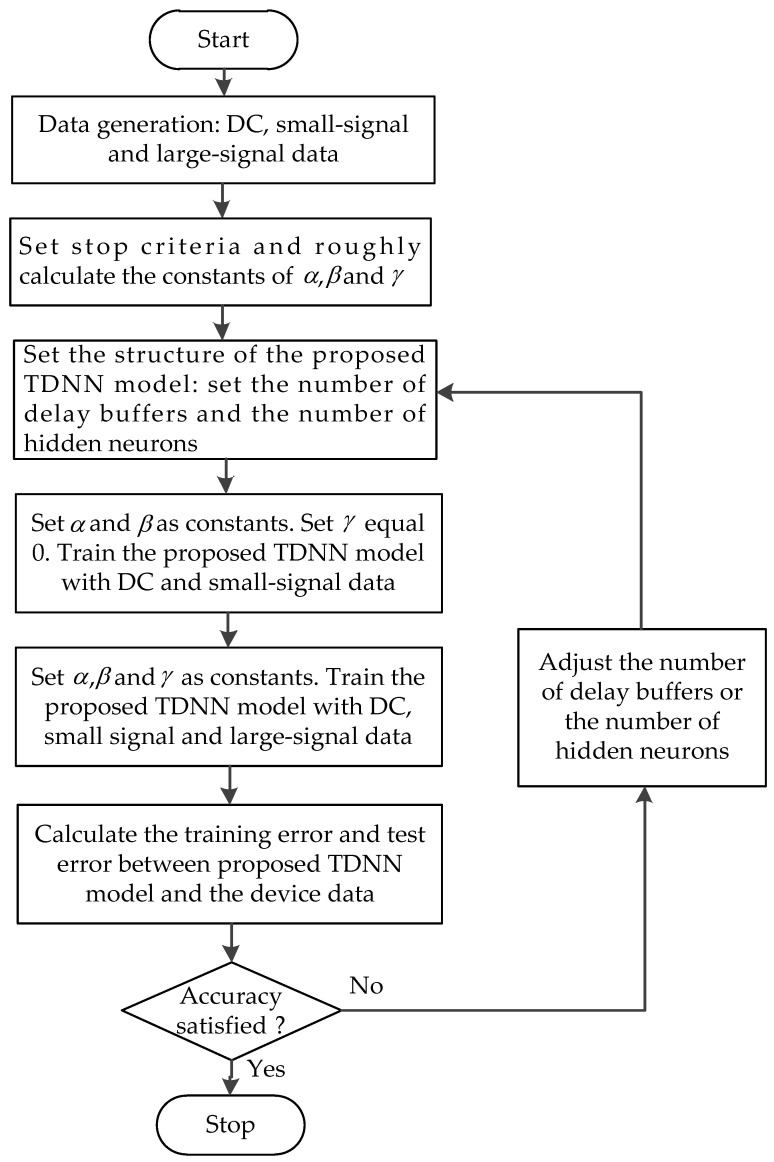
The process for the proposed TDNN model development.

The first step for developing the proposed TDNN model is to generate DC, small-signal and large-signal device data used for training and testing. The range of the training data should cover the range of the testing data. After data preparation, we have to determine the structure of the proposed TDNN model, including the number of delay buffers and the number of hidden neurons. After these preparation works, we begin to train the proposed TDNN model. In the beginning, the number of delays buffers can be tried from 1, i.e., *N_d_* = 1 and the hidden neurons can be tried with a smaller number. We first set α   and  β    as constant that are roughly decided by the value range of the training data and the number of DC data, and small-signal data, and set γ equals 0. The proposed TDNN model can be trained with DC and small-signal data by adjust the neural network weights according to the error back propagation algorithm. After the first step training (it may need hundreds or thousands times of iteration, which is according to the practical problem), α, β  and γ  will be set as constants. Subsequently, the proposed TDNN model can be trained combined with DC, small-signal S-parameters, and large-signal harmonic data. After this step training, the training error will be calculated. When it is less than *E_t_* (user defined error criteria), the process of the training will stop. After the overall training, a separate set of DC, small-signal and large-signal data called test data, which are never used in the training, is used to test the quality of the proposed TDNN model. The test error *E_Te_* is defined as the error between the model responses and the test data. If the test error is also lower than the threshold error *E_t_*, then the model training process terminates and the proposed TDNN model is ready to be used for high-level design. Otherwise, the overall model training process will go to the previous step being repeated with different numbers of hidden neurons or different numbers of delay buffers. Figure 2 shows the flowchart illustrating the overall development process of the proposed TDNN model.

## 4. Examples

### 4.1. GaAs Metal-Semiconductor-Field-Effect Transistor (MESFET)

In this example, the TDNN method is used to model a Keysight advanced design system (ADS) [32] internal GaAs MESFET device [33] with the Statz model. Table 1 shows the parameters for the Statz model in ADS. We perform DC, small-signal, and large-signal training together in NeuroModelerPlus [34]. Training data includes DC data at different DC points, S-parameter data at different biases and large-signal harmonic data generated at different fundamental frequencies (1–6 GHz), input power levels (−5–7 dBm), and loads (40–60 Ohm), as seen in Table 2. The training data set and test data set are not randomly divided shown in Table 2. There are DC data at 162 different DC points, bias-dependent S-parameter data at 120 different biases, and harmonic data at a total of 936 combinations of input power, fundamental frequency, and load for training data. There are DC data at 130 different DC points, bias-dependent S-parameter data at 95 different biases, and harmonic data at a total of 120 combinations of input power, fundamental frequency, and load for test data. All of the training data was generated in ADS after performing DC simulation, S-parameter simulation, and harmonic balance simulation for getting DC, S-parameter, and harmonic data, respectively. The range of *V_g_* and *V_d_* in DC case can cover the range of *V_g_* and *V_d_* in small-signal S-parameter and harmonic cases. The frequency range of S-parameter data can cover the frequency range of harmonic data which is calculated by the fundamental frequency with the number of harmonics considered in the harmonic modeling process. The range of test data is within the range of training data. In this example, we choose the time delay parameter of the TDNN as 0.0045 ns. We perform the training for the proposed TDNN technique according to part 3. The proposed TDNN model is built after nearly 3000 iterations of DC and small-signal training and 300 iterations of DC, small-signal, and large-signal training. It takes roughly 1.5 h with the Intel core i9-9900 CPU at 3.60 GHz of the computing system. When the training is finished, we compare the accuracy of the proposed TDNN model at different training conditions shown in Table 3.

**Figure 3:**
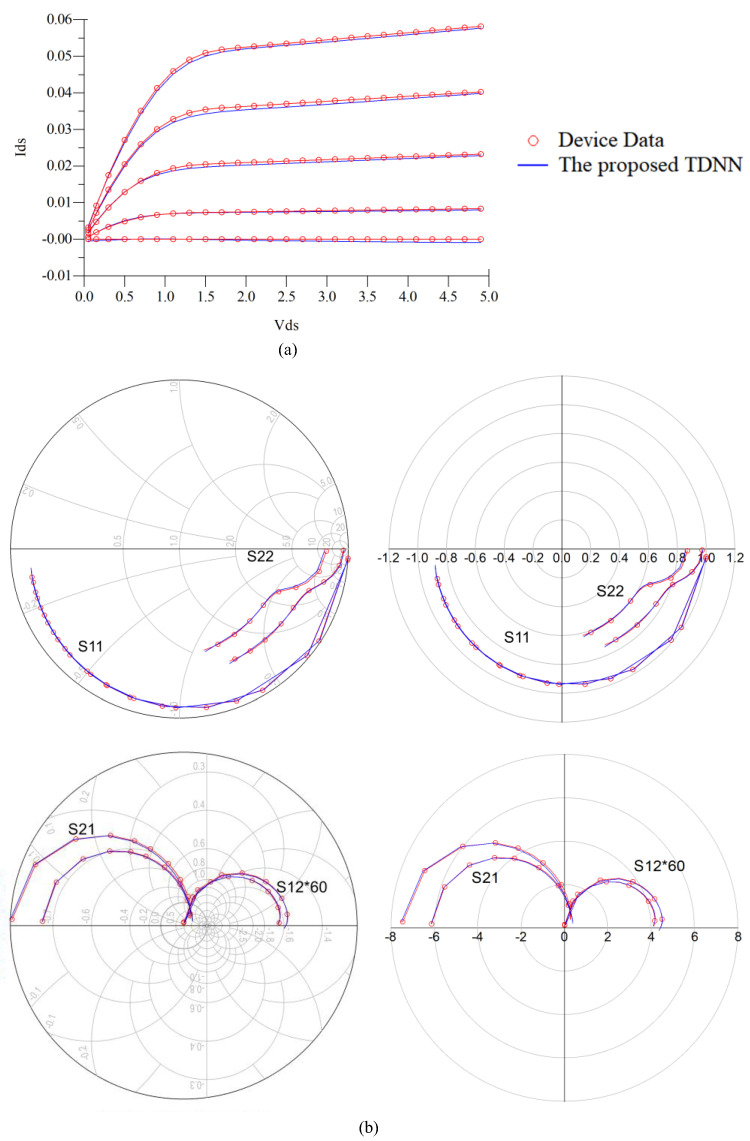
For GaAs metal-semiconductor-field-effect transistor (MESFET) example, comparison of DC and S-parameters at multiple biases of the device data and the proposed TDNN model. (**a**) DC. (**b**) S-parameters at two test biases of (−0.3 V, 3.6 V) and (0.1 V, 2.1 V). The DC and S-parameters shown in the figure from proposed TDNN is test data which is never used in the training. The frequency range of S-parameters for this MESFET example is from 0.1 GHz to 40.1 GHz.

**Figure 4:**
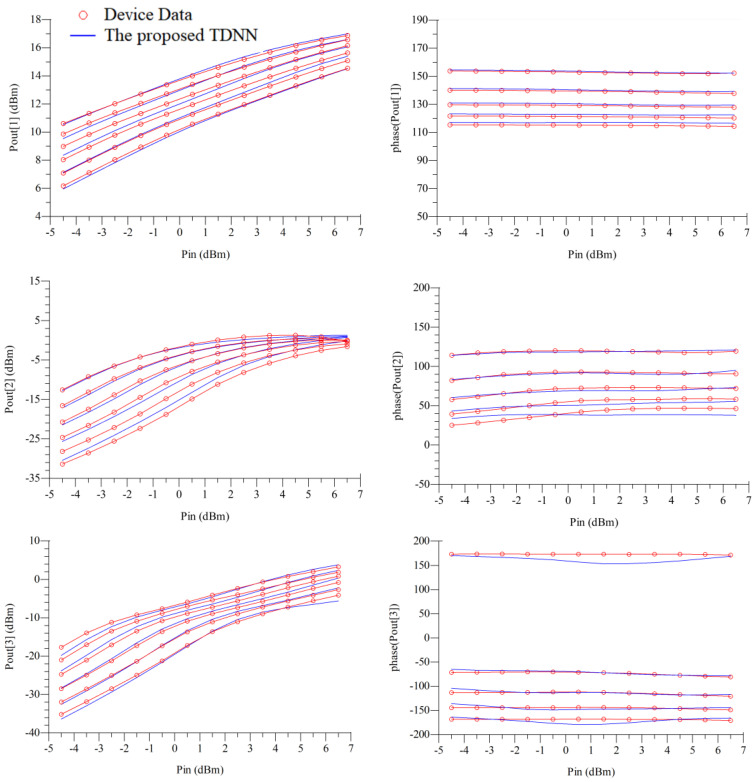
Comparison of the harmonic balance (HB) responses between the proposed TDNN model and the device data at the test load: 45 Ω, the fundamental frequency points: from 1.5 to 5.5 GHz, test bias: (*V_g_*: −0.15 V, *V_d_*: 3.1 V), and input power levels: from −4.5 to 6.5 dBm in the MESFET example.

For comparison purpose, we also developed the static model using the MLP technique for this GaAs MESFET example. MLP is a feedforward neural network. The inputs of the MLP and TDNN both are *V_g_* and *V_d_* of the transistor, the outputs of the MLP and TDNN are both *I_g_* and *I_d_* of the transistor. For fairly comparison, we both use a three-layer MLP for MLP technique and the *f_ANN_* of the TDNN technique, the activation functions are both the sigmoid function, the numbers of hidden neurons for these two techniques are both same, and the learning algorithm used in this paper is quasi-newton method. We compare the results from the MLP model and the proposed TDNN model that is shown in Table 3. In the case of DC, S-parameter at multiple biases, and HB training, the TDNN approach has accuracy advances over the static modeling technique, as seen in Table 3. This is because nonlinear devices usually contain dynamic effects, which is not adequate for device modeling by using the static modeling technique (MLP). However, when compared with MLP (only contains the present information), the proposed TDNN includes not only present information, but also the history information, which is necessary for nonlinear device modeling, especially when nonlinear device contains dynamic effects. When the number of delay buffers increases, the error of the proposed TDNN model when compared with device data decreases rapidly. We choose the condition (*N_d_* = 4, training error = 2.38%, test error = 1.88%) in Table 3 to present the results of our proposed TDNN model. DC, S-parameters, and HB responses of the proposed TDNN model are shown in Figure 3 and Figure 4. Finally, in this proposed TDNN model, the number of hidden neurons is 40, time delay parameter of the TDNN is 0.0045 ns, and the number of delay buffers is 4. From these figures, we can see the proposed TDNN model can accurately model the nonlinear microwave device.

### 4.2. GaAs High-Electron Mobility Transistor (HEMT)

**Figure 5:**
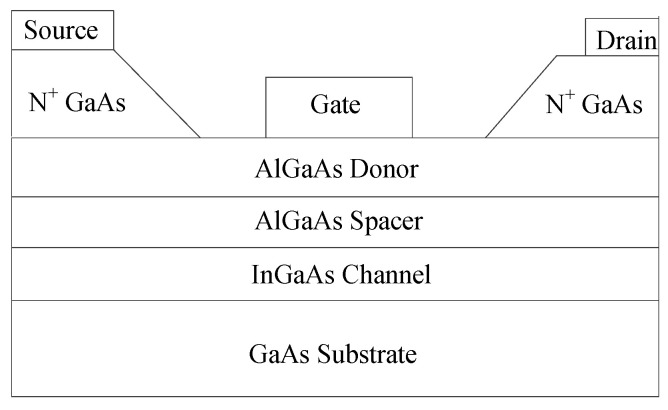
The structure of the high-electron mobility transistor (HEMT) device in Medici simulator used for data generation.

In this example, the proposed TDNN method is used to model the GaAs HEMT device. Training and test data were generated from a five-layer GaAs-AlGaAs-InGaAs HEMT example given in a physics-based device simulator Medici [35]. The structure of the HEMT [36] used in setting up the physical-based simulator is shown in Figure 5. Table 4 shows the parameters for the HEMT device. We performed DC, small-signal, and large signal training of the proposed TDNN according to the algorithm in part 3 with NeuroModelerPlus [34]. Training data includes DC data at different DC points, S-parameter data at different biases and large-signal harmonic data generated at different fundamental frequencies (2–5 GHz) and input power levels (−20–10 dBm), as seen in Table 5. The static bias is chosen as: *V_g_*: 0.2 V and *V_d_*: 5 V. The training data set and test data set are not randomly divided shown in Table 5. There are DC data at 378 different DC points, bias-dependent S-parameter data at 138 different biases, and harmonic data at a total of 44 combinations of input power, fundamental frequency, and load for training data. There are DC data at 310 different DC points, bias-dependent S-parameter data at 110 different biases, and harmonic data at a total of 33 combinations of input power, fundamental frequency, and load for test data. All of the training data was generated in Medici after performing DC simulation, S-parameter simulation, and harmonic balance simulation for getting DC, S-parameter, and harmonic data, respectively. The range of *V_g_* and *V_d_* in DC case can cover the range of *V_g_* and *V_d_* in small-signal S-parameter and harmonic cases. The frequency range of S-parameter data can cover the frequency range of harmonic data, which is calculated by the fundamental frequency with the number of harmonics considered in the harmonic modeling process. The range of test data is within the range of training data. In this example, we choose time delay parameter of the TDNN as 0.005 ns. After nearly 2000–3000 iterations of DC and small-signal training and 300 iterations of DC, small-signal, and large-signal training, the proposed TDNN model is built. It takes roughly 1.5–2 h with the Intel core i9-9900 CPU at 3.60 GHz of the computing system. When the training is finished, we compare the accuracy of the proposed TDNN model at different training conditions shown in Table 6.

**Figure 6:**
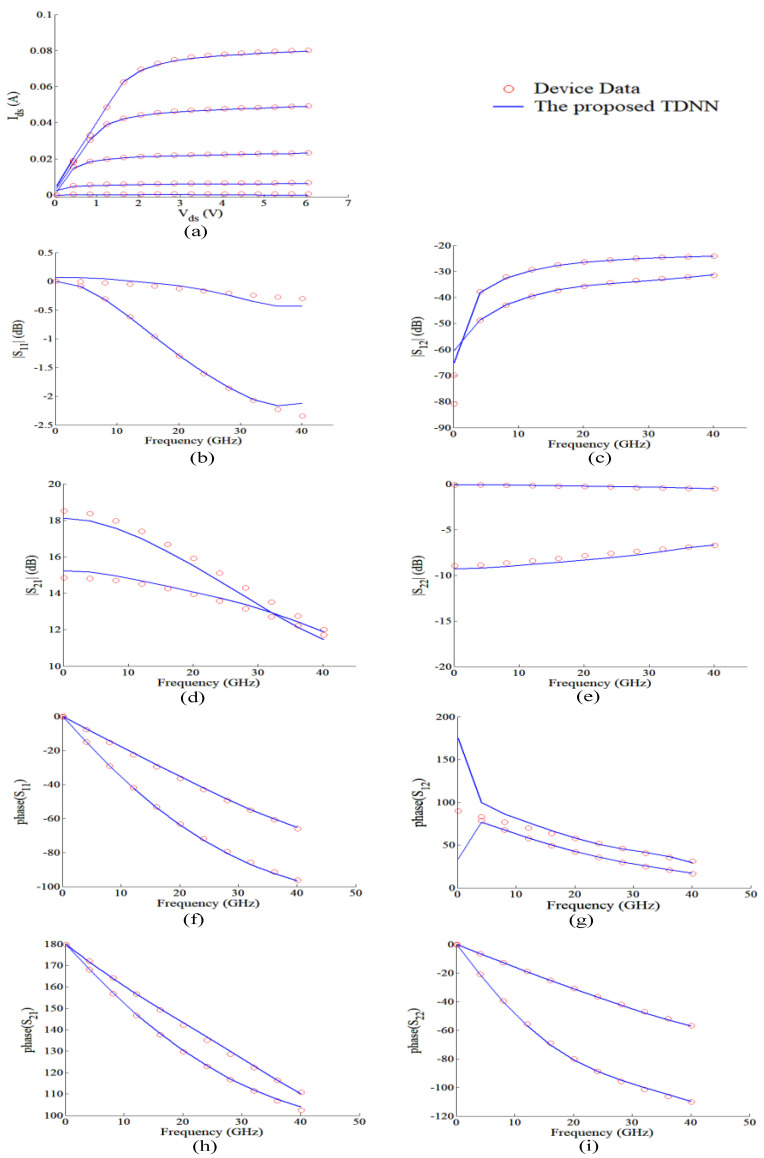
Comparison between the proposed TDNN model and the device data using DC and S-parameters at multiple biases for the HEMT example. (**a**) DC. (**b**–**i**) Magnitudes and Phases of S-parameters at two test biases of (*V_g_* and *V_d_*) at (0.1 V, 5.6 V) and (0.7 V, 2.1 V). The DC and S-parameters shown in the figure from proposed TDNN is test data which is never used in the training.

**Figure 7:**
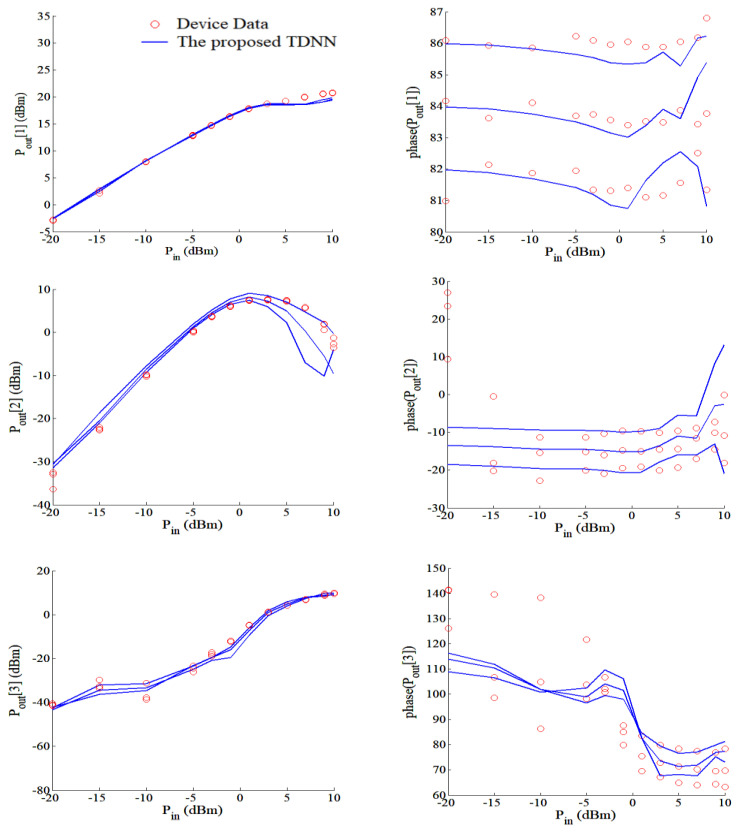
Comparison of magnitude and phase responses of the proposed TDNN model and device data at fundamental frequency 2.5 GHz, 3.5 GHz, and 4.5 GHz. Each blue line represents the magnitude or phase of output power along the input power at different fundamental frequencies.

For comparison purpose, we have also developed MLP model for this GaAs HEMT example. The inputs of the MLP and TDNN are *V_g_* and Vd of the transistor, the outputs of the MLP and TDNN are *I_g_* and *I_d_* of the transistor. For fairly comparison, we both use a three-layer MLP for MLP technique and the *f_ANN_* of the TDNN technique, the activation functions are both the sigmoid function, the numbers of hidden neurons for these two techniques both are same, and the learning algorithm used in this paper is quasi-Newton method. In the complicated case, DC, S-parameter at multiple biases and HB training together, TDNN model has huge accuracy advantage over MLP model, as seen in Table 6. In this table, the error of the TDNN model compared with test data reduces as the number of delay buffers increases. When comparing the number of hidden neurons 30 and 40, we can see as the number of hidden neurons increases, the accuracy enhances slowly. We choose the condition (*N_d_* = 7, 40 hidden neurons, training error = 1.15%, and test error = 1.9%) in Table 6 in order to present the results of our proposed TDNN model. The DC, S-parameters and HB responses of the proposed TDNN model are shown in Figure 6 and Figure 7. Finally, in the proposed TDNN model for this GaAs HEMT example, the number of hidden neurons is 40, the time delay parameter of the TDNN is 0.005 and the number of delay buffers is 7. From these figures, we can see that the proposed TDNN technique can accurately model the GaAs HEMT example.

## 5. Conclusions

In this paper, we have proposed a TDNN based technique for nonlinear microwave devices modeling. We have proposed a set of new formulations for training with DC, small-signal and large-signal data. We also have proposed an algorithm for the proposed TDNN model development. The modeling of GaAs MESFET and GaAs HEMT examples has successfully demonstrated that the TDNN based technique can accurately build nonlinear microwave device models. Using measurements to validate the comparison with real situation could be a useful direction. In the future direction, the thermal and trapping effects can be combined into the proposed TDNN. In the future, conventional device modeling method as compared with proposed TDNN can be a useful direction. As a potential future direction, the proposed TDNN technique can be investigated for other semiconductor technologies, such as Si and GaN based FETs. In the future, modeling and design microwave absorbers by the proposed technique can also be investigated.

## Figures and Tables

**Table 1:** Parameters for Statz model.

Parameter Name	Vaule	Parameter Name	Vaule
*Cgs* (F)	9.581 × 10^13^	*Lambda* (1/V)	0.05
*Cgd* (F)	7.598 × 10^14^	*Alpha* (1/V)	3.0
*Cds* (F)	1 × 10^14^	*B* (none)	3.0
*Crf* (F)	1 × 10^14^	*Rgd* (Ohm)	3
*Vto* (V)	0.5	*Rg* (Ohm)	1
*Beta* (A/V^2^)	0.310	*Rd* (Ohm)	5
*Vbi* (V)	0.9	*Rs* (Ohm)	2

**Table 2:** Training data and test data for GaAs metal-semiconductor-field-effect transistor (MESFET).

Data Type	Parameter Name	Training Data			Test Data		
		Min	Max	Step	Min	Max	Step
DC data	*V_g_* (V)	−0.6	0.4	0.2	−0.5	0.3	0.2
	*V_d_* (V)	0 0.4	0.2 5	0.1 0.2	0.05 0.3	0.15 4.9	0.1 0.2
Small-signal data	*V_g_* (V)	−0.6	0.4	0.2	−0.5	0.3	0.2
	*V_d_* (V)	0 0.4 2.6	0.2 2.2 5	0.1 0.2 0.4	0.05 0.3 2.4	0.15 2.1 4.8	0.1 0.2 0.4
	*f* (GHz)	0.1	40.1	1	0.1	40.1	1
Large-signal data	*V_g_* (V)	−0.2	−0.1	0.1	−0.15	−0.15	0
	*V_d_* (V)	3.0	3.2	0.2	3.1	3.1	0
	Pin (dBm)	−5	7	1	−4.5	6.5	1
	freq (GHz)	1	6	1	1.5	5.5	1
	Load (Ohm)	40	60	10	45	55	10

**Table 3:** Accuracy comparison of two modeling approach at different conditions.

Approach	Training	Test
MLP	53.99%	51.14%
TDNN (*N_d_*= 1)	6.16%	6.22%
TDNN (*N_d_*= 2)	3.59%	2.75%
TDNN (*N_d_*= 3)	2.95%	2.11%
TDNN (*N_d_*= 4)	2.38%	1.88%

**Table 4:** Values of geometrical/physical parameters for high-electron mobility transistor (HEMT) device.

Parameter Name		Value (um)
Gate Length (um)		0.2
Gate Width (um)		100
Thickness (um)	AlGaAs Donor Layer	0.025
	AlGaAs Spacer Layer	0.01
	InGaAs Channel Layer	0.01
	GaAs Substrate	0.045
Doping Density (1/cm^3^)	AlGaAs Donor Layer	1 × 10^18^
	InGaAs Channel Layer	1 × 10^2^
	Source N+	2 × 10^20^
	Drain N+	2 × 10^20^

**Table 5:** Training data and test data for GaAs HEMT.

Data Type	Parameter Name	Training Data			Test Data		
		Min	Max	Step	Min	Max	Step
DC data	*V_g_* (V)	−0.2	0.8	0.2	−0.1	0.7	0.2
	*V_d_* (V)	0	6.2	0.1	0.05	6.15	0.1
Small-signal data	*V_g_* (V)	−0.2	0.8	0.2	−0.1	0.7	0.2
	*V_d_* (V)	0 0.4 2.6	0.2 2.2 6.2	0.1 0.2 0.4	0.05 0.3 2.4	0.15 2.1 6.0	0.1 0.2 0.4
	*f* (GHz)	0.1	40.1	1	0.1	40.1	1
Large-signal data	Pin (dBm)	−20 −3	−5 9	5 2	−20 −3	−5 9	5 2
	freq (GHz)	2	5	1	2.5	4.5	1

**Table 6:** Accuracy comparison from different training conditions.

Approach	30 Hidden Neurons		40 Hidden Neurons	
	Training Error	Test Error	Training Error	Test Error
MLP	31.13%	33.98%	33.07%	34.11%
TDNN (*N_d_*= 1)	6.41%	6.58%	6.24%	6.48%
TDNN (*N_d_*= 3)	3.10%	3.32%	2.68%	2.88%
TDNN (*N_d_*= 5)	2.44%	2.51%	2.16%	2.24%
TDNN (*N_d_*= 7)	1.49%	1.86%	1.15%	1.9%
